# Crystal structure of rare earth and group III nitride alloys by ab initio calculations

**DOI:** 10.1038/s41598-020-73405-5

**Published:** 2020-10-02

**Authors:** Maciej J. Winiarski, Dorota A. Kowalska

**Affiliations:** Institute of Low Temperature and Structure Research, Polish Academy of Sciences, ul. Okólna 2, 50-422 Wrocław, Poland

**Keywords:** Semiconductors, Electronic properties and materials, Structure of solids and liquids, Electronic properties and materials, Semiconductors

## Abstract

The ground state phases of ternary alloys of rare earth and group III nitride semiconductors have been investigated within the density functional theory. The most energetically favorable crystal phases among possible cubic and hexagonal structures, i.e., the rock salt, zinc blende, wurtzite, and hexagonal BN, were determined. The type of a unit cell and the lattice parameters of the materials are presented as a function of their composition. Furthermore, effects of strain on ground states of group III and rare earth nitride materials are discussed. The findings presented in this work discloses the wurtzite type materials as being stable with relatively low contents of rare earth elements. It is expected that the wurtzite phase will be very persistent only in the La-based systems. Nevertheless, the two-dimensional hexagonal atomic layers are revealed as being a metastable phase for all alloys studied. This finding supports the conclusion of previous experimental reports for Sc-doped GaN systems that the presence of rare earth ions in group III nitride materials leads to flattening of the wurtzite type layers.

## Introduction

Band gap ($E_g$) engineering in group III nitrides was recently achieved *via* alloying with ScN and YN. The wurtzite (WZ) ${\text {Al}}_{1-x} {\text {Sc}}_x {\text {N}}$, ${\text {Ga}}_{1-x} {\text {Sc}}_x {\text {N}}$, and ${\text {Al}}_{1-x} {\text {Y}}_x {\text {N}}$ materials were experimentally obtained^1–7^. Such alloys exhibit a linear dependence of $E_g$ on composition, which differentiates them from group III nitride alloys^8^. The density functional theory (DFT) based investigations of electronic structures of Sc- and Y-doped AlN and GaN considered the WZ and zinc blende (ZB) type systems as candidate materials for applications in optoelectronics^9–16^.

Solid solutions of rare earth (*RE*) and group III nitrides are expected to adopt a hexagonal ground state for relatively high *RE* contents. It is because of a hypothetical metastable phase of the hexagonal BN-type, which existence was experimentally indicated, i.e., the WZ layers of ${\text {Ga}}_{1-x} {\text {Sc}}_x {\text {N}}$ are flattened in a vicinity of Sc atoms^2^.The recent studies showed that in ${\text {Al}}_{1-x} {\text {Sc}}_x {\text {N}}$ thin films, deposited by magnetron sputter epitaxy on sapphire, WZ-structure is favored up to *x* = 0.41^6^. The rock salt (RS) phase is preferred in systems with high Sc contents, prepared on cubic MgO^17^.

The DFT-based investigations of strain effects on structural properties of ScN, YN, LaN, and LuN suggest a possibility of hexagonal BN phase in (Sc,Y,Lu)N systems with unit cells having strongly increased volumes^18^. This finding is supported by the previous considerations of the metastable hexagonal phase in ScN^19^. Interestingly, the WZ phase collapses to the BN structure in the Sc-,Y-, and Lu-bearing systems, whereas it is the most energetically favorable in strained LaN. Because the ZB phase in *RE*N materials is unfavorable in general, one may expect that solid solutions of these systems with group III nitrides may adopt one of the WZ, BN, and RS ground state structures.

In this work, the structural properties of solid solutions of *RE*N and group III nitride semiconductors are investigated with the DFT-based methods. The ground state phases are obtained *via* analysis of the total energy of various possible phases of these systems (WZ, BN, ZB, and RS). The effects of hydrostatic and biaxial strain on total energy of the materials are further considered. The theoretical findings presented in this work are discussed with respect to the available experimental data for Sc- and Y-doped AlN and GaN materials.

**Figure 1:**
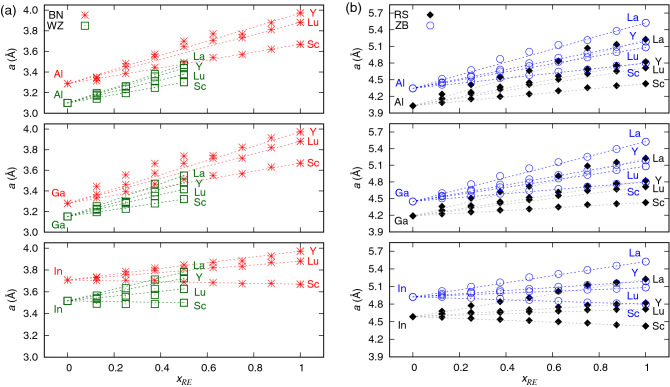
Lattice parameters *a* of (**a**) wurtzite and hexagonal, and (**b**) rock salt and zinc blende phases of ternary alloys of group III and rare earth nitrides.

## Results and discussion

Structural properties of hexagonal phases of ternary solid solutions of group III and *RE* nitrides are depicted in Fig. 1a. Except ${\text {In}}_{1-x} {\text {Sc}}_x {\text {N}}$ alloys, the lattice parameters of materials studied here increase with increasing content of *RE* ions, which reflects their generally bigger ionic radii when compared with those of group III ions^20^. In turn, the strong difference in the hexagonal lattice parameters *a* of WZ- and BN-type materials is connected with the *c/a* ratios of these structures, i.e., the 2D structure of BN exhibits smaller distances between the atomic layers with respect to those present in the 3D WZ phase. The latter one is transformed into the BN-type structure with flat atomic layers during the process of full structural relaxation for materials with $x_{RE}$ higher than 0.5. On one hand, a transition between the WZ and BN phases could lead to a step-like change in *a* and *c/a* in such systems. On the other hand, the WZ-like atomic layers in samples of ${\text {Ga}}_{1-x} {\text {Sc}}_x {\text {N}}$ are strongly flattened in the vicinity of Sc ions in general^2^. Conditions promoting the growth of 2D hexagonal *RE*N materials require further experimental investigations.

Some deviations from the linear Vegard’s law dependence of *a* on $x_{RE}$ are found for the BN-type systems with strong variations of the ionic radii of components. This effect is related to the flat shape of the 2D hexagonal layers, which prevents a full relaxation of atomic positions in alloys. One may expect a relatively small lattice mismatch in the WZ-type In-based materials deposited on InN, whereas Ga- and Al- based thin films may be feasible to obtain on ZnO substrate.

According to Fig. 1b, the dependences of cubic lattice parameters *a* on *RE* contents reveal a linear character. Although the stability of La-containing systems is questionable due to the strong lattice mismatch between group III nitrides and LaN, a small lattice mismatch between the mixed group III/*RE* materials and cubic *RE*N substrates seems feasible to obtain. They may also be grown on the ZB group III nitrides, which exhibit significantly bigger lattice parameters *a* then their hypothetical RS counterparts.

**Figure 2:**
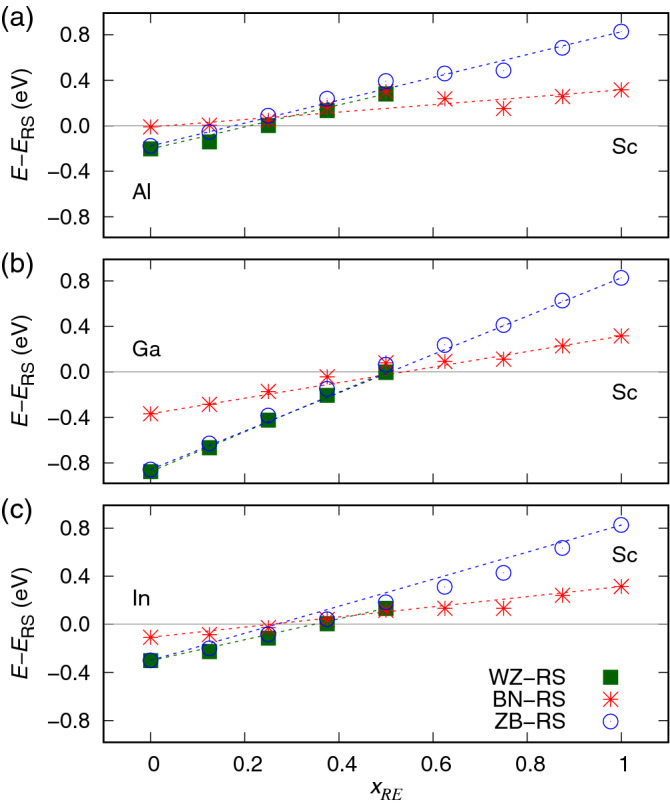
Total energy of wurtzite, hexagonal, and zinc blende phases with respect to the rock salt equilibrium phase of ScN, calculated for (**a**) ${\text {Al}}_{1-x} {\text {Sc}}_x {\text {N}}$, (**b**) ${\text {Ga}}_{1-x} {\text {Sc}}_x {\text {N}}$, and (**c**) ${\text {In}}_{1-x} {\text {Sc}}_x {\text {N}}$ alloys.

**Figure 3:**
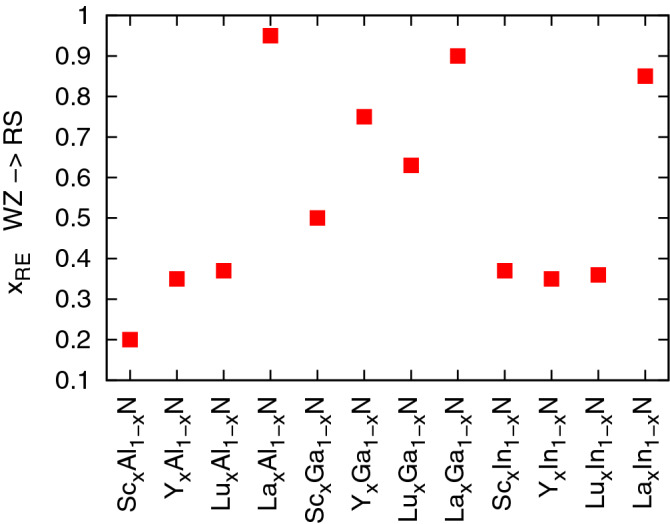
The compositions required for the wurtzite to rock salt phase transition in ternary alloys of group III and rare earth nitride semiconductors.

**Figure 4:**
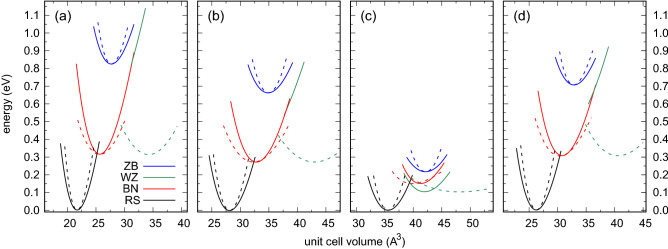
Energy–volume curves of rock salt, hexagonal, wurtzite, and zinc blende phases under hydrostatic (solid lines) and biaxial strain (dashed lines) calculated for (**a**) ScN, (**b**) YN, (**c**) LaN, and (**d**) LuN.

The total energies of WZ-, BN-, and ZB-type unit cells of (Al;Ga;In)$_{1-x} {\text {Sc}}_{x} {\text {N}}$ semiconductors, relative to the RS ground state of ScN, are presented in Fig. 2. According to these results, the WZ phase is energetically favorable for Sc contents below 0.2, 0.5, and 0.4 for Al-, Ga-, and In-based alloys, respectively. Systems with higher *x* are expected to exhibit the RS ground state. The WZ layers are completely flattened in the materials with *x* above 0.5, i.e., they are transformed into the BN-type ones. The energies of the BN-type systems are close to those of the WZ ones for *x* in the WZ-RS transition region. The total energy of the hexagonal BN-type phase of alloys indicate this phase as metastable in materials with *x* above 0.5, for which the WZ phase is not present and the ZB phase is strongly unfavorable. Regarding the existence of the ZB phase, which is almost degenerate with the WZ ground state in the parent AlN, GaN, and InN compounds, it becomes less favorable with increasing Sc content in the solid solutions. Despite the similarity in dependencies of total energy on the unit cell content for all materials, it is noticeable that the GaN-based systems with low Sc contents reveal the highest energy differences between particular phases. In addition, the WZ phase is the most persistent with increasing Sc content.

It is worth recalling that the recent experimental studies reported high quality ${\text {Al}}_{1-x} {\text {Sc}}_x {\text {N}}$ materials with *x* up to 0.41^6^, whereas the samples of ${\text {Ga}}_{1-x} {\text {Sc}}_x {\text {N}}$ were obtained for *x* lower than 0.3^1,7^. The results presented in this work are generally consistent with the experimental findings mentioned above. Any possible discrepancies may be attributed to the fact that the process of sample growth is a complex issue, i.e, various materials may be obtained depending on growing conditions. According to the total energy dependence on Sc content in alloys, depicted in Fig. 2a, the Al-based systems may adopt the metastable WZ phase for *x* up to 0.5. This phase should also be feasible to obtain in Ga-based alloys for similar *x*, as inferred from Fig. 1b. However, the optimal growing conditions for particular compositions of alloys and possible effects related to strain are unknown. The correspondence between the LDA-derived results presented in this work and the mixing enthalpies within the general gradient approximation (GGA) reported for ${\text {Al}}_{1-x} {\text {Sc}}_x {\text {N}}$^3^ may be further considered. Namely, the differences in total energy of hexagonal and cubic phases in the Al-rich regime, obtained in our calculations, are slightly smaller with respect to the GGA-derived results. This observation suggests a lower *x* required for transition between the WZ and RS phases.

The dependences of total energies of the cubic and hexagonal phases calculated for Y, La, and Lu-bearing alloys based on group III nitride materials (not shown here) are very similar to those discussed above for Sc-doped systems. The contents of *RE* ions required for a transition between WZ and RS phases in all alloys considered here are summarized in Fig. 3. It can be noticed that the Ga-bearing systems may form WZ-type alloys for higher $x_{RE}$ than Al- and In-based materials. Interestingly, the solid solutions of LaN with group III nitrides may adopt the WZ phase for almost all compositions. The RS phase is only expected to be a ground state in materials with very high contents of La. A tendency to form the WZ-type structure in La-based nitrides was also suggested in the previous theoretical investigations for the LaN parent compound^18^.

The WZ phase in alloys with high *RE* content is surprisingly robust. The relationship between a tendency to form flat hexagonal layers by *RE*N, which was confirmed in experimental studies^2^, and the WZ type structure of group III nitrides is puzzling. As depicted in the energy–volume curves in Fig. 4, this phase is expected to be completely unstable in *RE*N systems in hydrostatic conditions. However, the strong biaxial strain may stabilize the metastable WZ structure in these materials. The energies of the BN and WZ phases are comparable in such a case. It is worth noting that the metastable WZ phase in LaN is energetically preferable with respect to the BN one in all conditions considered here. These finding supports the results presented in Fig. 3, i.e., a very wide range of $x_{RE}$ in systems with the WZ-type structure.

**Figure 5:**
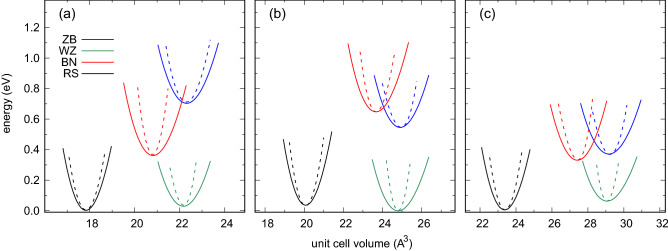
Energy–volume curves of rock salt, hexagonal, wurtzite, and zinc blende phases under hydrostatic (solid lines) and biaxial strain (dashed lines) calculated for (**a**) ${\text {Al}}_{0.75} {\text {Sc}}_{0.25} {\text {N}}$, (**b**) ${\text {Ga}}_{0.5} {\text {Sc}}_{0.5} {\text {N}}$, (**c**) ${\text {In}}_{0.625} {\text {Sc}}_{0.375} {\text {N}}$.

The influence of hydrostatic as well as biaxial strain on structural properties of Sc-bearing alloys close to the RS–WZ transition may be inferred from Fig. 5. The total energies of WZ and RS phases of these systems are comparable. One may expect both structures to exist as metastable phases in *RE*-doped group III nitrides. In such WZ systems, compressive strain may cause flattening of the hexagonal atomic layers, which eventually leads to their transition into the 2D graphene-like layers. The energy-volume curves depicted in Fig. 5 reflect the energy differences between the phases, as discussed for the data in Fig. 2. The existence of ZB phase in the materials studied is impossible because the WZ one is energetically favorable in the particular ranges of unit cell volumes. The effect of biaxial strain on structural properties of the materials is expected to be strong. The transition between the hexagonal phases considered may be induced by nonhydrostatic strain connected with a relatively small change in the unit cell volume.

Similar findings were obtained for ternary alloys of group III nitrides with other *RE*N (not shown in this work). Namely, the presence of *RE* ions in such solid solutions causes three general effects. The first one is diminishing of the ZB phase with simultaneous tendency to form the BN type flat atomic layers. The second one is a possibility of the RS phase formation which is infeasible in pure group III nitride materials. The third one is eventual use of strain as a tool for the intentional design of structural properties of solid solutions of *RE*N and group III nitrides.

## Conclusions

The results of DFT-based calculations suggest a tendency to form the RS phase for relatively low contents of *RE* ions in solid solutions of *RE*N and group III nitrides. The La-bearing systems are exceptions, in which the WZ phase is expected to be robust. However, the local structure of hexagonal group III nitrides doped with *RE* ions is rather flattened with respect to the ideal WZ one due to the fact that *RE*N materials tend to adopt the BN-like phase. Only strong nonhydrostatic strain may stabilize the WZ-type phase in these systems. In general, some nonequilibrium conditions may be employed for stabilization of hexagonal phases in *RE*-doped group III nitrides being close to the WZ–RS transition. Nevertheless, the RS ground state in *RE*N materials doped with group III elements is undoubted. The findings presented in this work should encourage further experimental research on electronic and structural properties of mixed *RE* and group III nitrides.

**Figure 6:**
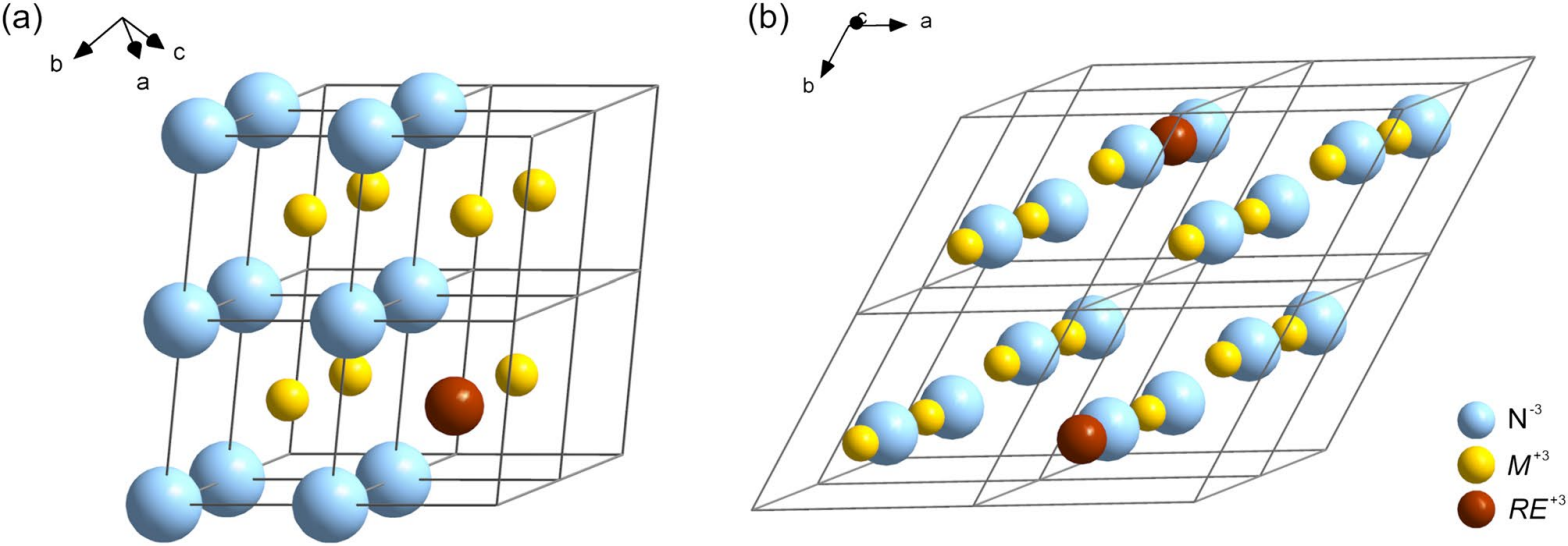
The cubic and hexagonal supercells of $M_{1-x}RE_{x} {\text {N}}$ material, where *M* = {Al; Ga; In} and *RE* = {Sc; Y; La; Lu} ions occupy the same position and here *x* = 0.125, employed in this work.

## Methods

The DFT-based calculations have been performed with the use of the Abinit package^32,33^. The plane augmented wave (PAW) atomic datasets were taken from the JTH table^34^. The Perdew–Wang parameterization of an exchange–correlation functional within the local density approximation (LDA) was employed^35^. The solid solutions were modeled with $2 \times 2 \times 2$ supercells of hexagonal (WZ, BN) and cubic (RS, ZB) unit cells, containing 16 and 32 atoms, respectively. The supercells used in this work are depicted in Fig. 6. The lattice parameters and positions of all atoms in the supercells were fully relaxed via stress/forces optimization. The volume–enegy curves were fitted to a Murnaghan-type equation of state^36^.

## Data Availability

The datasets analysed during the current study are available from the corresponding author on reasonable request.
